# Influence of two collection frequency intervals on sperm quality of standard and miniature bull Terriers during short breeding periods: A clinical field study

**DOI:** 10.14202/vetworld.2024.820-828

**Published:** 2024-04-15

**Authors:** J. Salvado, D. Catilina, P. Borges, J. Simões, A. Martins-Bessa

**Affiliations:** 1 Department of Veterinary Science, Integrated Master of Veterinary Medicine, University of Trás-os-Montes and Alto Douro, Portugal; 2 Prime Veterinary Practice, Chesterfield, United Kingdom; 3 Faculty of Veterinary Medicine, Lusófona University, Lisbon, Portugal; 4 CECAV-Animal and Veterinary Research Center, University of Trás-os-Montes and Alto Douro, Vila Real, Portugal; 5 AL4AnimalS-Associate Laboratory for Animal and Veterinary Sciences, Vila Real, Portugal

**Keywords:** bull terriers, dog, ejaculate, ejaculatory frequency, sperm quality

## Abstract

**Background and Aims:**

The quality of canine sperm can be influenced by many factors, such as breed, body weight, age, ejaculatory frequency, nutrition, and environment. In the UK, it is common practice for standard Bull Terriers (SBT) and miniature Bull Terriers (MBT) to require male donors during a short breeding period. The aim of this study was to evaluate the effect of semen collection frequency on ejaculate volume and nine sperm parameters in SBT and MBT males, considering age and body condition score (BCS).

**Materials and Methods:**

Ejaculates from six adult SBTs and four MBTs were collected 5 times at two consecutive intervals (Time Series [TS]1, 24 h *vs*. TS2, 48 h), 1 week apart. Ejaculate volume, concentration, total output, viability (live sperm), subjective total motility, vigor, and total morphological defects, including head, midpiece, and tail defects of sperm, were evaluated. A multivariable mixed linear model for repeated measures was used to analyze the effects of semen collection frequency, age, breed, and BCS on ejaculate volume and sperm parameters.

**Results:**

Semen collection frequency, age, and, to a lesser extent, breed, and BCS significantly affected sperm parameters. Semen collection frequency affected all sperm parameters (p < 0.05) but not ejaculate volume (p > 0.05). Total sperm output, sperm vigor, total motility, and tail defects decreased (p < 0.05) at the end of TS1. However, sperm parameters remained relatively constant (p > 0.05) in TS2 between semen collection sessions. Overall, poorer sperm parameters were observed in older dogs (aged 5–8 years) than in younger dogs (aged 4 years). MBT produced less (p < 0.001) ejaculate volume (3.2 ± 0.2 mL *vs*. 4.3 ± 0.2 mL: Least Squares Mean ± Standard Error of Mean), lower total sperm output (221.8 ± 19.2 × 10^6^ vs. 348.6 ± 19.2 × 10^6^) and lower total morphological defects (25.0 ± 1.1% *vs*. 31.3 ± 0.9%), and a higher percentage of live sperm (77.0 ± 1.4% *vs*. 71.7 ± 1.1%) than SBT. In addition, a BCS of 4 positively influenced (p < 0.05) viability, vigor, and total sperm motility.

**Conclusion:**

Despite differences in age, breed, and BCS, better sperm parameter values were observed in all semen collection sessions. However, intensive semen collection (TS1) appears to be less effective in maintaining good sperm quality. For breeding or artificial insemination purposes, a 48-h interval between collection sessions is recommended for both breeds. The results of this study could be used to further optimize assisted reproductive technologies in both breeds.

## Introduction

Dog sperm quality can be influenced by many factors, such as age, size, genetic factors, nutrition, management, housing conditions, and environment [1]. Frequency of semen collection remains one of the most important factors affecting sample quality, as described previously in several species such as stallions [2], rams [3], bulls [4], alpacas [5], and dromedary camels [6]. Similar studies in dogs are scarce.

In French bulldogs, semen collection at 24 h intervals resulted in an earlier decrease in total ejaculate volume, sperm concentration, vigor, and normal morphology than at 48 h intervals [7]. However, dual semen collection within 1-h intervals in dogs does not appear to have any detrimental effects on sperm parameters, and both collections can increase the total number of sperm cells [8] and a reduction in bacterial contamination in the second ejaculate [9]. However, frequent collection is expected to result in more morphological abnormalities and reduced sperm concentration and motility [10, 11]. It is recommended that the interval between semen collections should not be more than 2–5 days [11] to preserve semen quality.

To the best of our knowledge, no data have been reported in the scientific literature regarding the evaluation of the frequency of semen collection in standard Bull Terriers (SBT) and miniature Bull Terriers (MBT) breeds. Females tend to synchronize their estrus cycle, known as the “dormitory effect” [12], and it is common to use the same dog to breed various females in a short period. In addition, in the UK, a number of show dogs are used daily for mating within a short period of time, with successful female pregnancy outcomes.

We conducted the present clinical field study to assess the impact of semen collection frequency on sperm quality for two successive semen collection intervals, namely, 24 and 48 h, which were conducted 1 week apart in the SBT and MBT breeds. Furthermore, the influence of body condition score (BCS) and age on semen parameters was evaluated.

## Materials and Methods

### Ethical approval

This study was approved by the Institutional Review Board of the University of Trás-os-Montes and Alto Douro (protocol code DOC_FP.22423-847PA63057) and conducted in accordance with the Declaration of Helsinki.

### Study period and location

This study was conducted from September to October 2021, in Chesterfield, Derbyshire and Sheffield, South Yorkshire, United Kingdom.

### Animals, local environment, and study design

Ten adult male dogs (six SBT and four MBT) aged between 1 and 8 years were used. All dogs were registered with the Bull Terrier Kennel Club and were clinically healthy at the time of collection, with both testes in the scrotum and no known pathologies. All dogs, except for two SBTs, had previously sired a litter. The breed, age, weight, BCS [13], and previous fertility of the dogs were recorded before each collection.

All dogs included in the study underwent semen collection at their owners’ homes located within the Derbyshire and Peak District regions of England, United Kingdom. Written informed consent was obtained from each owner for the collection and evaluation of semen and its use in the publication of the study results. The reproductive rest period was approximately 3 months, with ejaculates collected 2 weeks after the completion of the dog competition season. We obtained ejaculates only for the purposes of this study.

We divided the study into two consecutive semen collection regimens for each breed (SBT and MBT). Semen was collected once daily at approximately the same hour for 5 consecutive days in Time Series (TS)1. All dogs were collected 5 times after a week-long break, with a 48-h interval between each collection (TS2). In this study, 100 semen samples were collected and evaluated for standardized parameters.

### Ejaculate collection and sperm evaluation

For male stimulation, swabs impregnated with bitch vaginal secretions were used. The first and second fractions of the ejaculate were collected jointly, and the third fraction was discarded after collection. A disposable plastic bag (Minitube®, UK) with a 15 mL tube attached to its end was used for ejaculate collection. The ejaculate color (clear, milky, or other) and volume (mL) were evaluated immediately after collection.

Nine sperm parameters were evaluated: sperm concentration (×10^6^/mL), total sperm output (volume × sperm concentration; ×10^6^), viability (live sperm %), vigor (scale of 0–5), total motility (%), total morphological defects (%), and head, midpiece, and tail defects (%). Sperm concentration was measured using a photometer (Spermacue® SDM1 Minitube®, UK), and the total sperm output was calculated.

For each sperm sample, two smears were prepared and stained with eosin-nigrosin and Spermac® stain (Spermac Stain Kit, Minitube®, Barcelona, Spain) to evaluate the live sperm (sperm live/dead ratio) and sperm morphology, respectively. A total of 200 sperm cells were evaluated for each staining method at 1000× magnification using an Olympus CX23 light microscope (Olympus, Tokyo, Japan).

Total motility (%) and vigor (0–5) were subjectively assessed using a contrast-phase microscope (Motic® BA400, Motic, Xiamen, China) based on the vigor of the sperm motility: 0, none; 1, very weak; 2, weak; 3, intermediate; 4, strong; and 5, very strong. A minimum of four 7.5 μL drops of non-diluted semen samples were used for total motility and vigor evaluation. Total motility (%) was assessed based on the percentage of sperm moving in the entire microscopic field, as observed at 400× magnification. Multiple microscopic fields (>10) were evaluated. Vigor was scored from 0 to 5 on the basis of motile sperm speed of movement. To minimize variability in sperm evaluation, ejaculates were collected and evaluated throughout the study by the same researcher with over 5 years of experience in this field.

To assess sperm morphology, a drop of semen was placed at one end of a microscope slide and then carefully drawn to the opposite end, allowing it to air dry completely. Smears were prepared according to the manufacturer’s instructions. Sperm morphological defects, including head, midpiece, and tail abnormalities, were classified according to the affected spermatic region, allowing the determination of the percentage of morphologically normal sperm [14]. Sperm morphology was independently evaluated blindly by a different researcher than the one who evaluated motility.

### Statistical analysis

Differences in weight and age between the SBT and MBT groups were evaluated using the Student’s t-test. Animals with a BCS of 3–4 out of 5 points were classified as 3 (<3.5) or 4 (≥3.5) points.

A multivariable model (standard least squares personality) for repeated measures was constructed according to the following equation to test the effect of the independent variables on each semen parameter:

Yijomp=Hi+Lj+Bo+Sp+tmi+eijomp

where:

Yijomp is a vector of all observations and is represented by the least squares value.

Hi is the fixed effect for breed (2 levels: SBT *vs*. MBT).

Lj is the fixed effect for each session (10 levels: 1–10 successive semen collections).

Bo is the fixed effect for age (2 levels: 1–4 *vs*. 5–8 years old).

Sp is the fixed effect for BCS (2 levels: 3 *vs*. 4 points).

tmi is the random effect for animal (m) within the collection session and

eijomp is a vector for residuals.

Linear mixed models were fitted using the restricted maximum likelihood method. Differences between groups were evaluated using the Student’s t test. In addition, second-degree polynomial correlations were established to predict different sperm parameters according to age (1–8 years), independent of the number of semen collection sessions.

Data were analyzed using JMP® 16 software for Windows (SAS Institute®, Cary, NC, USA). Results are presented as least square mean ± standard error of the mean. The significance level was set at 0.05 for all analyses, and 0.5 > p ≤ 0.1 was considered statistically significant.

## Results

### Animals

The average weight for SBT and MBT was 31.3 ± 3.2 kg and 13.3 ± 1.5 kg, respectively (p < 0.001). No differences in age were observed between SBT and MBT (4.2 ± 1.1 years and 4.3 ± 1.4, respectively; p > 0.05).

### Global effect of modulated variables

Overall, sperm quality parameters were mostly affected by semen collection session (TS) and age and to a lesser extent by breed and BCS (Table-1).

**Table-1:** Effect of breed, age, body condition score, and collection session on sperm quality parameters.

Parameter	Breed	Age	Body condition	Collection session
Volume (mL)	***	p = 0.07	NS	NS
Sperm concentration (×10^6^)	NS	***	p = 0.08	p = 0.06
Total sperm output (×10^6^)	***	***	p = 0.06	***
Live sperm (%)	**	***	*	***
Motility (%)	NS	***	*	**
Vigor (0–5 scale)	NS	**	*	***
Total morphological defects (%)	***	*	NS	***
Head defects (%)	NS	NS	NS	***
Mid-piece defects (%)	NS	***	*	p = 0.09
Tail defects (%)	***	NS	NS	***

NS=Non-significant,

* p < 0.05,

** p < 0.01,

*** p < 0.001

### Semen collection session (TS) effect

In this study, pairwise comparisons were made between the collection sessions (n = 10), where sessions 1–5 corresponded to TS1 and sessions 6–10 corresponded to TS2 (Tables-2 and 3). Total sperm output and total sperm motility decreased (p = 0.05) during TS1 compared with sessions 1 and 2. Similarly, sperm vigor decreased in session 5 compared with that in sessions 1, 2, and 3 (p = 0.05). In contrast, these sperm parameters remained constant during TS2, and the difference between sessions 6 and 10 was not statistically significant (p > 0.05).

**Table-2:** Effect of ejaculate collection frequency on ejaculate volume and five sperm parameters (LSM ± SEM).

Time Serie	Collection session	Volume of ejaculate (mL)	Sperm concentration (×10^6^/mL)	Total sperm output (×10^6^)	Alive sperm (%)	Vigor (0–5 scale)	Total motility (%)
TS1	1	3.6 ± 0.4^a^	100.0 ± 16.3^a^	362.9 ± 70.8^a^	62.8 ± 3.1^a^	3.9 ± 0.3^a,b,c^	75.0 ± 4.5^a,b,c^
	2	3.7 ± 0.5^a^	77.0 ± 7.9^a,b^	275.9 ± 37.1^a,b,c^	68.8 ± 3.5^a,b^	3.7 ± 0.2^b,c,d^	76.0 ± 2.3^a,b^
	3	3.7 ± 0.5^a^	59.2 ± 8.5^b^	203.7 ± 35.2^c,d^	72.2 ± 2.3^b,c^	3.7 ± 0.2^c,d^	67.0 ± 3.1^c,d^
	4	3.9 ± 0.5^a^	64.2 ± 14.2^b^	224.1 ± 35.8^b,c,d^	72.6 ± 2.1^b,c^	3.2 ± 0.3^d,e^	69.0 ± 3.2^b,c,d^
	5	3.2 ± 0.5^a^	58.9 ± 10.6^b^	170.1 ± 28.0^d^	74.3 ± 2.1^b,c,d^	2.9 ± 0.2^e^	64.0 ± 3.7^d^
TS2	6	3.6 ± 0.6^a^	86.7 ± 9.5^a,b^	305.4 ± 50.2^a,b^	73.8 ± 4.6^b,c^	4.3 ± 0.3^a^	75.2 ± 5.1^a,b,c^
	7	3.9 ± 0.5^a^	84.5 ± 7.4^a,b^	315.1 ± 42.5^a,b^	79.5 ± 3.0^c,d^	4.1 ± 0.2^a,b,c^	72.0 ± 4.6^a,b,c,d^
	8	4.4 ± 0.2^a^	81.1 ± 9.1^a,b^	349.9 ± 39.2^a^	81.4 ± 2.8^d^	4.2 ± 0.3^a,b^	75.5 ± 2.8^a,b,c^
	9	3.7 ± 0.4^a^	86.6 ± 8.9^a,b^	316.8 ± 40.6^a,b^	78.1 ± 3.1^c,d^	4.3 ± 0.2^a^	78.5 ± 2.7^a^
	10	4.1 ± 0.3^a^	80.7 ± 6.8^a,b^	327.0 ± 33.4^a^	79.5 ± 3.0^c,d^	4.1 ± 0.2^a,b,c^	79.0 ± 2.3^a^

^a,b,c,d^different superscript letters in the same column regarding all 10 semen collection sessions: p *<* 0.05. TS1=Time series 1, TS2=Time series 2, LSM=Least squares mean, SEM=Standard error of mean

**Table-3:** Effect of ejaculate collection frequency on sperm morphological defects (LSM ± SEM).

Time serie	Collection session	Total morphological defects (%)	Head defects (%)	Mid piece defects (%)	Tail defects (%)
TS1	1	34.0^a,b^	9.0^a^	5.9^a^	19.1^a^
	2	36.0^a^	4.5^b,c^	6.5^a^	25.0^b^
	3	28.9^a,b,c^	4.2^b,c^	6.6^a^	18.1^a,c^
	4	29.2^a,b,c^	3.7^b,c^	8.6^a,b^	16.9^a,c,d^
	5	29.1^a,b,c^	5.0^b^	10.7^b^	13.5^d^
TS2	6	27.0^a,b,c^	4.9^b^	6.6^a^	15.6^a,c,d^
	7	25.6^b,c^	3.7^b,c^	7.0^a^	14.5^c,d^
	8	22.8^c^	2.6^c^	6.7^a^	13.5^d^
	9	24.4^c^	3.8^b,c^	6.6 ^a^	14.0^c,d^
	10	25.2^b,c^	4.2^b,c^	7.1^a^	13.9^c,d^

^a,b,c,d^different superscript letters in the same column regarding all 10 semen collection sessions: p *<* 0.05. TS1=Time series 1, TS2=Time series 2, LSM=Least squares mean, SEM=Standard error of mean

In contrast, sperm vitality (live %) outcomes in session 1 (TS1) were poor (p < 0.05) compared with those in sessions 3–10 (TS1 and TS2). A higher percentage of head defects was observed in session 1 than in the remaining nine sessions (p < 0.05). Similar trends were observed for tail defects (Table-3).

No significant differences **(**p > 0.05) in ejaculate volume were observed between session collections, with an average volume of 3.9 ± 0.5 mL (p = 0.81). However, differences in sperm concentration were observed (p = 0.06). This tendency was attributed to the higher (p < 0.05) sperm concentration observed in session 1 compared with the other sessions in TS1. Furthermore, a tendency (p = 0.09) was observed for midpiece defects, with a higher (p = 0.05) value in session 5 than in all other sessions, except session 4 (Table-3).

### Age effect

A lower ejaculate volume was found in younger dogs (4.0 ± 0.2 mL) than in older dogs (3.5 ± 0.2 mL) (p = 0.07). As shown in Table-4, dogs aged 1–4 years had higher sperm concentration (p < 0.001), total sperm output (p < 0.001), live sperm (p < 0.001), sperm vigor (p < 0.01), total sperm motility (p < 0.001), and lower morphological defects (p = 0.05), including lower midpiece defects (p < 0.001) than older dogs aged 5–8 years.

**Table-4:** Effect of age on the ejaculate volume and sperm parameters (LSM ± SEM).

Parameter	Age (years old)		p-value

	1–4	5–8	
Volume of ejaculate (mL)	4.0 ± 0.2	3.5 ± 0.2	0.07
Sperm concentration (×10^6^ mL)	89.7 ± 4.4	66.1 ± 4.5	< 0.001
Total sperm output (×10^6^)	334.0 ± 16.4	236.2 ± 16.7	< 0.001
Alive sperm (%)	77.8 ± 1.2	71.0 ± 1.2	< 0.001
Vigor (0–5 scale)	4.1 ± 0.2	3.6 ± 0.2	0.002
Sperm Motility (%)	78.6 ± 1.4	67.8 ± 1.4	< 0.001
Total morphological defects (%)	26.8 ± 0.9	29.5 ± 1.0	0.05
Head defects (%)	4.2 ± 0.3	4.9 ± 0.3	0.16
Mid piece defects	5.7 ± 0.5	8.8 ± 0.5	< 0.001
Tail defects (%)	16.9 ± 0.7	15.9 ± 0.8	0.32

LSM=Least squares mean, SEM=Standard error of mean

**Figure-1:**
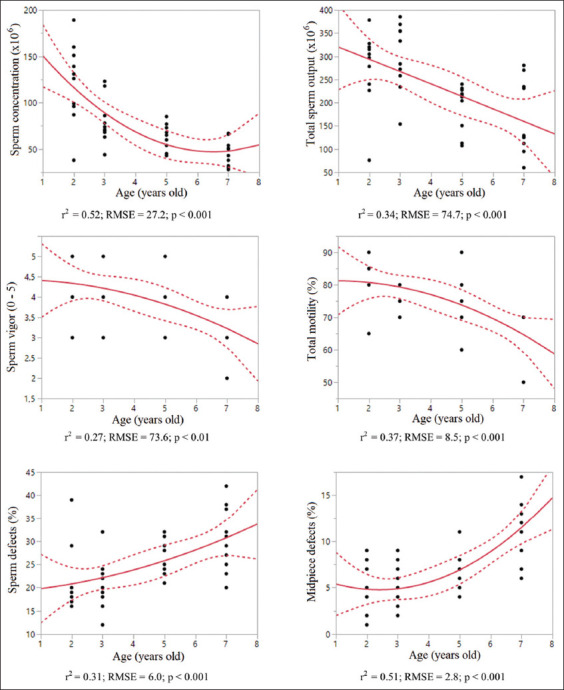
Prediction of spermatozoa parameters according to age, in Miniature bull terrier. Dashed lines=95% interval confidence, r^2^=Coefficient regression; RMSE=Root mean square error.

A 2-degree polynomial regression (Figure-1) predicted a progressive decrease in total sperm output or concentration and a progressive increase in total and midpiece defects as age increased in MBT. Age was responsible for 27% (lowest r^2^ = 0.27; sperm vigor; p < 0.001) to 52% (highest r^2^ = 0.52; sperm concentration; p < 0.001) of these variations. Interestingly, the percentage of live sperm did not show a correlation (p = 0.21) with increasing age; in fact, an even higher percentage was found in the 1–4-year age group.

**Figure-2:**
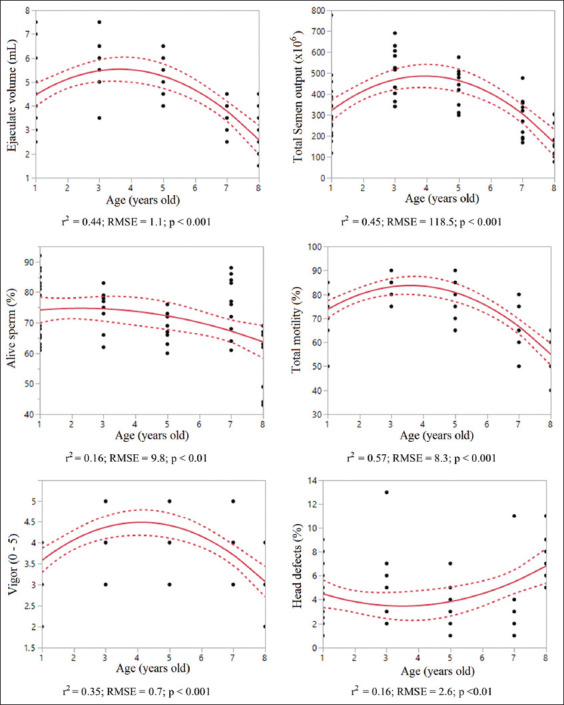
Prediction of spermatozoa parameters according to age in Standard bull terrier. Dashed lines=95% interval confidence, r^2^=Coefficient regression, RMSE=Root mean square error.

The highest predicted sperm parameter values (total sperm output, total motility, and sperm vigor; p < 0.01) were observed in dogs aged 3–5 years (Figure-2). However, the percentage of live sperm gradually decreased in dogs aged up to 8 years. Although predictions of sperm concentration (p = 0.10) and total defect (p = 0.41) were not significant, the percentage of head defects was lowest at 2–4 years of age (p < 0.001). Variation in ejaculate volume could be predicted (p < 0.001) according to age in this breed.

### Breed and BCS effects

Compared with SBTs, MBTs produced a lower volume of ejaculate (p < 0.001), total sperm output (p < 0.001), total morphological defects (p < 0.001), and tail defects (p < 0.001) and a higher live sperm percentage (p = 0.005). Other sperm parameters remained similar between breeds (p > 0.05).

The percentage of live sperm, vigor, and total motility in dogs with a BCS of 4 was higher (p < 0.05) than that in dogs with a BCS of 3. Although the ejaculate volume and total sperm output were similar between the two groups, dogs with a BCS of 4 tended to have a higher sperm concentration (p = 0.08) and total sperm output (p = 0.07). All values are presented in Table-5.

**Table-5:** Effect of breed and BCS on the ejaculate volume and sperm parameters (LSM ± SEM).

Parameter	Breed		p-value	BCS (points)		p-value
	
	MBT	SBT		3	4	
Volume of ejaculate (mL)	3.2 ± 0.2	4.3 ± 0.2	< 0.001	3.8 ± 0.2	3.8 ± 0.2	0.95
Sperm concentration (×10^6^ mL)	74.1 ± 5.1	81.8 ± 4.1	0.27	71.9 ± 4.9	84.0 ± 4.4	0.08
Total sperm output (×10^6^)	221.8 ± 19.2	348.6 ± 19.2	< 0.001	261.4 ± 18.1	308.8 ± 16.6	0.07
Alive sperm (%)	77.0 ± 1.4	71.7 ± 1.1	0.005	72.3 ± 1.3	76.4 ± 1.2	< 0.05
Vigor (0–5 scale)	3.8 ± 0.1	3.8 ± 0.1	0.93	3.7 ± 0.1	4.0 ± 0.1	< 0.05
Sperm Motility (%)	73.3 ± 0.2	73.1 ± 0.1	0.91	70.5 ± 1.6	75.9 ± 1.4	< 0.05
Total morphological defects (%)	25.0 ± 1.1	31.3 ± 0.9	< 0.001	28.4 ± 1.0	27.9 ± 0.9	0.70
Head defects (%)	4.4 ± 0.4	4.7 ± 0.3	0.57	4.8 ± 0.4	4.3 ± 0.3	0.31
Mid piece defects	7.4 ± 0.6	7.1 ± 0.5	0.70	8.0 ± 0.5	6.5 ± 0.5	0.05
Tail defects (%)	13.2 ± 0.9	19.5 ± 0.7	< 0.001	15.7 ± 0.8	17.1 ± 0.7	0.21

MBT=Miniature bull terrier, SBT=Standard bull terrier, BCS=Body Condition Score, LSM=Least squares mean, SEM=Standard error of mean

## Discussion

Although the previous studies have investigated the impact of factors such as age, body weight [1, 15], and environmental differences [16] on the quality of fresh semen in dogs, studies on male dog fertility are relatively scarce, and comparative studies between different breeds are even more limited. To the best of our knowledge, no previous study has compared the effect of semen collection frequency on ejaculate volume and sperm quality between two popular dog breeds, SBT and MBT. As expected, our study found differences in several sperm parameters between the two breeds.

First of all, as expected, MBT produced less ejaculate volume and total sperm output per ejaculate than SBT. In a retrospective study [15], small-sized dogs (<15 kg) ejaculated a mean volume of 3.2 ±0.4 mL and total number of 309.6 ± 45.4 × 10^6^ spermatozoa, whereas medium-sized dogs (16–40 kg) ejaculated a mean volume of 4.2 ±0.3 mL and total number of 551.3 ±33.7 × 10^6^ spermatozoa. In the present study, it was not possible to distinguish between breeds and body weight.

The amount of ejaculate collected is influenced by various factors, including the practitioner who performs the collection and the environmental conditions that can affect the quality of the ejaculate. In this study, these factors were minimized by conducting sample collection in a quiet room with similar environmental conditions and using the same practitioner. The results of the present study are similar to those of previous studies conducted in a field setting, where it was not feasible to collect ejaculates before the start of the study. In addition, this study was limited because it only assessed subjective motility.

In the present study, MBTs had a higher concentration of live sperm than SBTs. Rijsselaere *et al*. [1] suggested that dogs with higher body weight tend to produce ejaculates with lower quality parameters, probably due to less efficient scrotal and testicular thermoregulation mechanisms.

Our results partially support this notion, as we compared two breeds with different body weights. However, our study revealed that MBTs had a higher percentage of tail defects due to total morphological defects than SBTs.

Moreover, MBTs and SBTs showed lower concentration values (100.8 ± 51.57 x 10^6^/mL at the first collection) than beagles (367 ± 159 × 10^6^/mL) [17] or multiple breed stud dogs (276 ± 233.51 × 10^6^/mL) [18]. In our study, we collected the first and second fractions of the ejaculate simultaneously, which decreased the sperm concentrations. However, when the sperm outputs were compared, the numbers were within the expected range, as previously described. Furthermore, sperm morphology values were within the range described for other breeds [18].

In contrast to human andrology, no guidelines have been established for the optimal timing of canine semen collection [14]. A recent study shows that collecting semen after a 1-day interval is more effective than after a 4-day interval [19].

The collection of sperm over a period of 5 consecutive days, as conducted in the TS1 stage of our study, appears to be less effective in completing the spermiogenesis process. A significant decrease in total sperm output was observed during the final collection session (session 5) compared to the other sessions of TS1, and this trend was also observed for sperm vigor and total motility. In addition, a higher percentage of midpiece defects was noted in session 5 than in sessions 1–3, whereas tail defects were lower. These findings suggest that sperm depletion and a lack of complete maturation of sperm may be due to the 24-h interval between collection sessions. Morphological secondary defects, which may have been acquired during sperm maturation, may also have played a role in these results, although the subtypes of sperm defects for each category were not reported in our study. Further, in-depth studies are required to investigate this aspect.

In contrast to TS2, the evaluated sperm parameters remained relatively stable between Sessions 6 and 10. In view of these results, it is recommended to maintain a 48-h interval between breeding and artificial insemination in these breeds. This suggestion agrees with the conclusion reported for the French Bulldog breed using a similar methodology: Five semen collection sessions (TS1 vs. TS2) with a 1-month rest period [7]. In their study, the volume, sperm concentration, vigor, and normal morphology of sperm were lower in the third, fourth, and fifth sessions of TS1 and only in the fourth and fifth sessions of TS2, compared to the first session of each TS. Total sperm motility decreased after the third and fourth sessions in TS1 and TS2, respectively.

Moreover, ejaculate volume was not significantly affected by the frequency of collection, contrary to previous research on French bulldogs (p < 0.05) [7]. However, further research on fertile dogs is required to confirm our findings. Similar results have been observed in boars collected 3 times a week (approximately every 48 h) and 7 times a week (daily) [20]. In boars, daily collection showed a decrease in sperm concentration, total sperm number per ejaculate, progressive motility, and morphological defects compared to collection every 3 days [20]. In our study, these sperm parameters remained constant between sessions, except for total sperm output and total motility in session 5, as previously reported.

Our results also suggest that male age is a crucial factor for sperm quality. In our study, younger dogs (1–4 years old) had better values for most sperm quality parameters (Table-5). Moreover, ejaculate volume was higher in younger dogs than in older dogs aged between 5 and 8 years.

This result is consistent with the results of several studies conducted in dogs and other domestic animals. For example, a study of different dog breeds revealed that males under 24 months of age ejaculated the highest number of sperm cells per collection, with higher sperm concentration, motility, and normal morphology [18]. Another study on male Great Danes [21] found lower motility values in dogs over 48 months of age, which can be attributed to the rapid aging process in large breeds.

Similarly, a previous study of different dog breeds of varying ages [22] showed an increase in the percentage of sperm with abnormal morphology, namely, sperm with cytoplasmic droplets, with increasing age. Moreover, age and total sperm count showed a negative correlation. Furthermore, another study showed that epididymal sperm quality decreased in senile dogs [23] and that male age was negatively correlated with epididymal sperm motility, sperm vigor, and viability. When epididymal function is reduced, sperm maturation also decreases.

In a recent study [24], the effects of age on sperm quality and its possible mechanisms in domestic animals were reviewed. The decline in sperm quality is related to a reduction in androgen levels and the number of germ cells, which directly affects spermatogenesis. In addition to decreased function of the epididymis and accessory glands, sperm DNA repair, seminal plasma, and sperm antioxidant protection have also decreased. These aspects are important in dogs because genetically valuable dogs are used for breeding even at an advanced age [24].

In addition, different studies on bulls have shown that age is also correlated with semen quality, specifically motility and total sperm number. The total sperm output increased from 3000 × 10^6^ sperm cells per collection, at approximately 12 months in the first collection, to 10,000 × 10^6^ sperm cells per collection between 27 and 30 months of age. During the same period, the groups showed the same tendency with respect to the total motility. Motility values of 70% and 76% were observed at 12 and 25 months of age, respectively, in the first collection [25]. More recently, Pardede *et al*. [26] observed that the total and progressive motility values of thawed semen decreased in older bulls aged 11–12 years compared with younger bulls aged 5–6 years (p < 0.01).

In this study, we used a quadratic polynomial function to estimate the sperm parameters for SBTs and MBTs based on age. There was a significant relationship between age and some of these variables, mainly weak to moderate regression coefficient, in both breeds. The quadratic effect of age was well evidenced by the generality of these sperm parameters (Figures-1 and 2). This non-linear relationship suggests that age is a crucial factor that partly accounts for fluctuations in some sperm parameters over time. In studies dividing dogs into young (1–3 years), middle (4–6 years), and senior (>7 years) age groups, sperm parameters vary according to age. However, differences in sperm parameters were not consistently significant among the three groups [27, 28].

Recent studies have also reported a negative influence of age on libido, motility, vigor, and morphology, which make sperm more susceptible to cryodamage [29, 30]. Aging reduces testicular blood flow and causes tissue damage [31]. Histologically, age has a negative impact on seminiferous tubules and increases testicular peritubular space fibrosis, contributing to a decrease in spermatogenic function [32].

In the current BCS scoring system [13], a score of 3–3.5 is typically considered ideal for dogs, while a score of 4.0–4.5 classifies them as overweight. In this study, dogs were classified based on a threshold of 3.5 points (3 *vs*. 4 points), and dogs with a BCS <3 or >4 points were excluded. Dogs with a BCS score of 3 had lower levels of live sperm, vigor, and total motility than those with a score of 4. In addition, the 3-point group showed a tendency toward higher sperm concentration and total sperm output than the other groups. These findings suggest that a BCS score between 3.5 and 4.0 is advantageous for reproductive success. However, previous studies have shown that overweight is becoming increasingly prevalent in dogs [33]. While a score of 3–3.5 is considered ideal for show dogs, a slightly improved BCS may be more advantageous for breeding. In view of the impact of BCS on fertility, it is important to ensure an ideal BCS before breeding [34].

### Limitations

This study has some limitations. First, semen was collected after a 3-month period of reproductive rest. All dogs used in this study were show dogs, and semen was collected 2 weeks after the end of the competition season to allow sufficient time to recover. Due to the limited availability of dogs, it was not feasible to collect them before the experiment, which may have partially influenced the TS1 result. Session 1 had a negative impact, as evidenced by the lower sperm viability and higher incidence of head defects compared to the other sessions. In the French Bulldog breed, the lowest percentage of morphologically normal sperm was also observed in the first session after a 1-month rest period [7].

Second, the subjectivity of sperm evaluation is a significant disadvantage. Although computer-assisted sperm analysis systems can provide automated and objective kinetic and morphological sperm analysis, manual examination is still considered the gold standard technique for evaluating morphological parameters [35]. In this study, the same experienced researcher performed all sperm parameter measurements to minimize errors caused by variable operators.

Finally, this study was conducted in a mobile reproductive laboratory under field conditions. Therefore, factors such as environmental climatization and unexpected events are less well controlled than in fixed reproductive laboratories. However, there were no significant unplanned incidents in this study.

## Conclusion

Total sperm output, vigor, total motility, and tail defects were lowest at the end of the TS1. However, the sperm parameter values remained constant throughout the semen collection sessions during the TS2 period. For breeding or artificial insemination purposes in MBT and SBT breeds, a 48-h interval between collection sessions is recommended.

The sperm parameters were affected by age, with the exception of head and tail defects, older dogs showed poor results. In addition, the ejaculate volume tended to be higher in younger dogs than in older dogs. However, there was a non-linear effect of age on some sperm parameters. Compared with SBTs, MBTs had a higher volume of ejaculate and total sperm output but lower sperm viability (live sperm%). The highest sperm viability, vigor, and total motility were observed in dogs with a BCS close to 4. These results can be used to further optimize assisted reproductive technologies in both breeds. However, further studies with larger sample sizes and more sophisticated methodologies are required to expand these findings in both breeds.

## Authors’ Contributions

JSa, DC, PB, and AMB: Conception and design of the study. JSi: Organized the database. JSa, JSi, and AMB: Defined the methodology and wrote the first draft of the manuscript. JSa and JSi: Performed validation and data analysis. All authors have read, reviewed, and approved the final manuscript.
